# Supplementary material for: Effects of Rare Phytocannabinoids on the Endocannabinoid System of Human Keratinocytes
Source: Int J Mol Sci. 2022 May 12;23(10):5430. doi: 10.3390/ijms23105430 (PMC9145865; doi:10.3390/ijms23105430)
Supplement: Supplementary file 1 [file ijms-23-05430-s001.zip › ijms-1713594-supplementary.pdf]

Effects of rare phytocannabinoids on the endocannabinoid system of human keratinocytes

Camilla Di Meo[1#], Daniel Tortolani[2#], Sara Standoli[1], Clotilde Beatrice Angelucci[3], Federico Fanti[1], Alessandro Leuti[2,4], Manuel Sergi[1], Salam Kadhim[5], Eric Hsu[5], Cinzia Rapino[3*] and Mauro Maccarrone[2,6*]

[1]*Faculty of Bioscience and Technology for Food Agriculture and Environment, University of Teramo, Teramo, 64100, Italy; cdimeo@unite.it (C.D.M.); sstandoli@unite.it (S.S.); ffanti@unite.it (F.F.); msergi@unite.it (M.S.)*

[2]*European Center for Brain Research (CERC)/Santa Lucia Foundation IRCCS, Rome, 00143, Italy; daniel.tortolani88@gmail.com*

[3]*Faculty of Veterinary Medicine, University of Teramo, Teramo, 64100, Italy; bcangelucci@unite.it*

[4]*Department of Medicine, Campus Bio-Medico University of Rome, Rome, Italy; a.leuti@unicampus.it*

[5]*InMed Pharmaceuticals Inc., Vancouver BC, V6C 1B4, Canada; ehsu@inmedpharma.com; skadhim@inmedpharma.com*

[6]*Department of Biotechnological and Applied Clinical Sciences, University of L'Aquila, 67100 L'Aquila, Italy*

[#]*Equally first authors.*

**Correspondence: crapino@unite.it (C.R.); mauro.maccarrone@univaq.it (M.M.)*

Supplementary Materials

Viability, apoptosis, and cell cycle of human HaCaT cells treated with different pCBs

CANNABIGEROL (CBG)

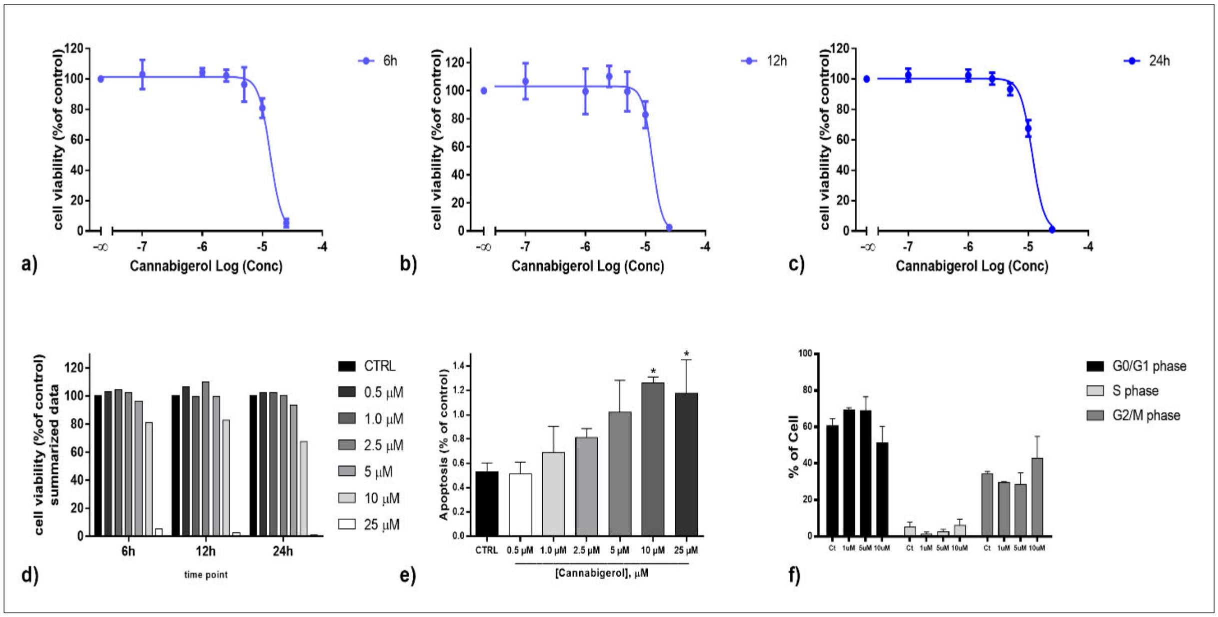

Figure S1. Viability, apoptosis, and cell cycle of human HaCaT cells treated with CBG. Cell viability of HaCaT cells treated with vehicle (CTRL) or increasing concentrations of CBG (0.5 μM, 1.0 μM, 2.5 μM, 5.0 μM, 10 μM, 25 μM), at the following time points: a) 6h, b) 12h, c) 24h. Values represent the mean ± SEM of three independent experiments (n = 3). d) Summary of cell viability data shown in panels a-c. e) Apoptosis of HaCaT cells treated with vehicle (CTRL) or increasing concentrations of CBG (0.5 μM, 1.0 μM, 2.5 μM, 5.0 μM, 10 μM, 25 μM) for 24h. f) Cell cycle analysis of HaCaT cells treated with vehicle (CTRL) or increasing concentrations of CBG (1.0 μM, 5.0 μM, 10 μM) for 24h. Values represent the mean ± SEM of three independent experiments (n = 3). Statistical analysis was performed by ONE-WAY ANOVA test followed by Bonferroni post hoc test. *P<0.05 *vs* CTRL.

CANNABICHROMENE (CBC)

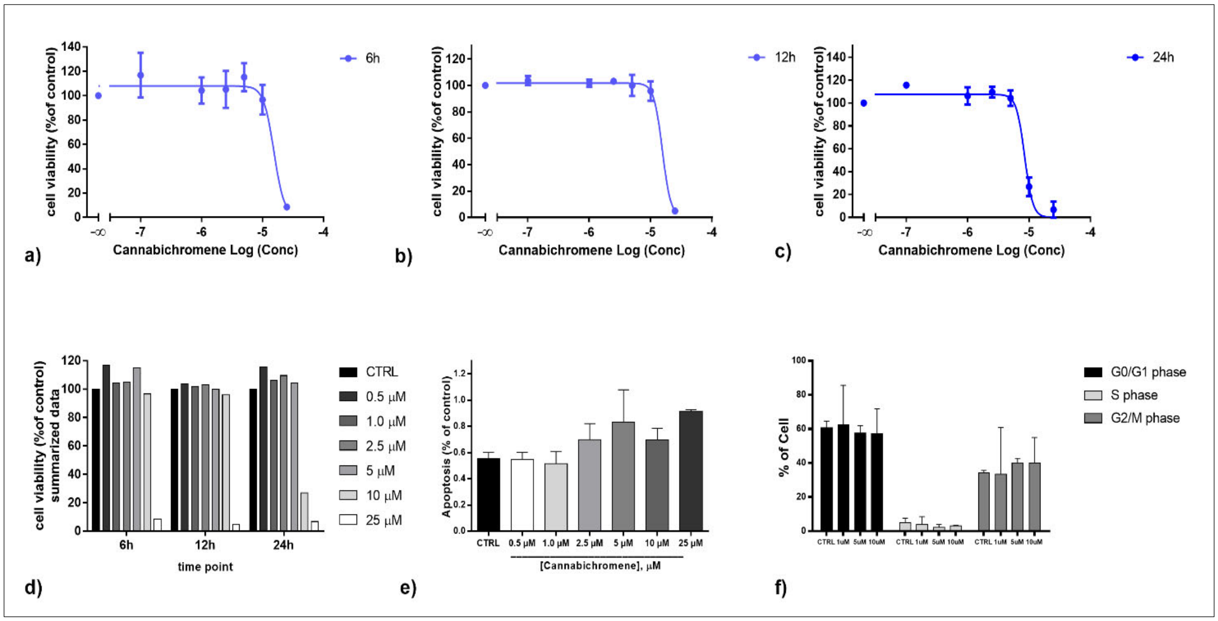

Figure S2. Viability, apoptosis, and cell cycle of human HaCaT cells treated with CBC. Cell viability of HaCaT cells treated with vehicle (CTRL) or increasing concentrations of CBC (0.5 μM, 1.0 μM, 2.5 μM, 5.0 μM, 10 μM, 25 μM), at the following time points: a) 6h, b) 12h, c) 24h. Values represent the mean ± SEM of three independent experiments (n = 3). d) Summary of cell viability data shown in panels a-c. e) Apoptosis of HaCaT cells treated with vehicle (CTRL) or increasing concentrations of CBC (0.5 μM, 1.0 μM, 2.5 μM, 5.0 μM, 10 μM, 25 μM) for 24h. f) Cell cycle analysis of HaCaT cells treated with vehicle (CTRL) or increasing concentrations of CBC (1.0 μM, 5.0 μM, 10 μM) for 24h. Values represent the mean ± SEM of three independent experiments (n = 3). Statistical analysis was performed by ONE-WAY ANOVA test followed by Bonferroni *post hoc* test.

TETRAHYDROCANNABIVARIN (THCV)

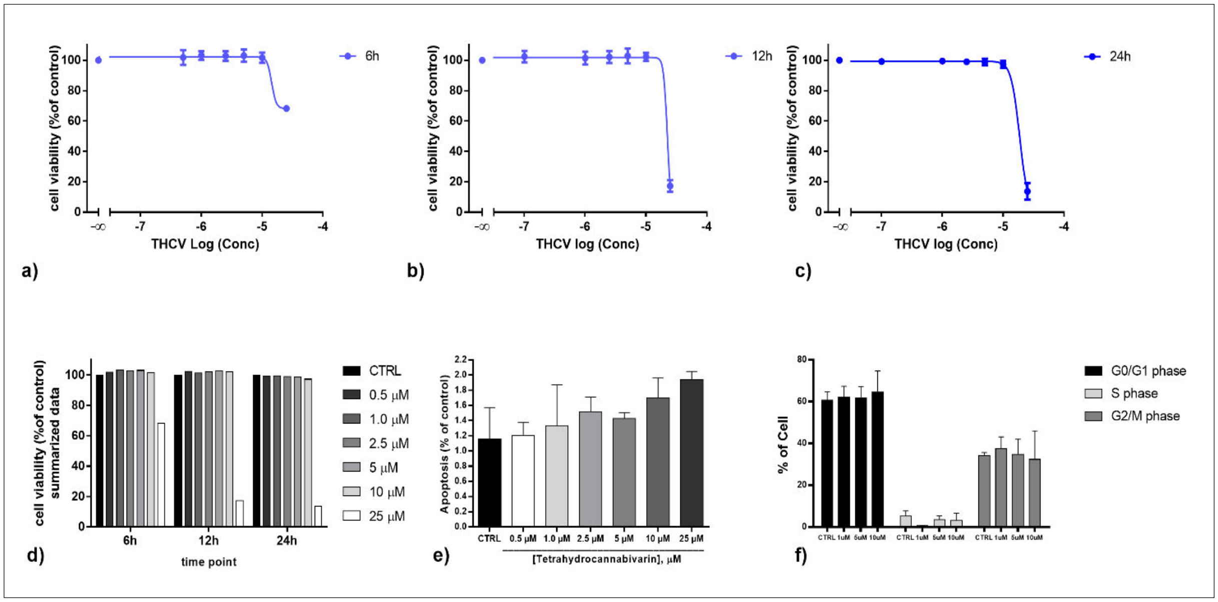

Figure S3. Viability, apoptosis, and cell cycle of human HaCaT cells treated with THCV. Cell viability of HaCaT cells treated with vehicle (CTRL) or increasing concentrations of THCV (0.5 μM, 1.0 μM, 2.5 μM, 5.0 μM, 10 μM, 25 μM), at the following time points: a) 6h, b) 12h, c) 24h. Values represent the mean ± SEM of three independent experiments (n = 3). d) Summary of cell viability data shown in panels a-c. e) Apoptosis of HaCaT cells treated with vehicle (CTRL) or increasing concentrations of THCV (0.5 μM, 1.0 μM, 2.5 μM, 5.0 μM, 10 μM, 25 μM) for 24h. f) Cell cycle analysis of HaCaT cells

treated with vehicle (CTRL) or increasing concentrations of THCV (1.0 μM, 5.0 μM, 10 μM) for 24h. Values represent the mean ± SEM of three independent experiments (n = 3). Statistical analysis was performed by ONE-WAY ANOVA test followed by Bonferroni *post hoc* test.

CANNABIGEROLIC ACID (CBGA)

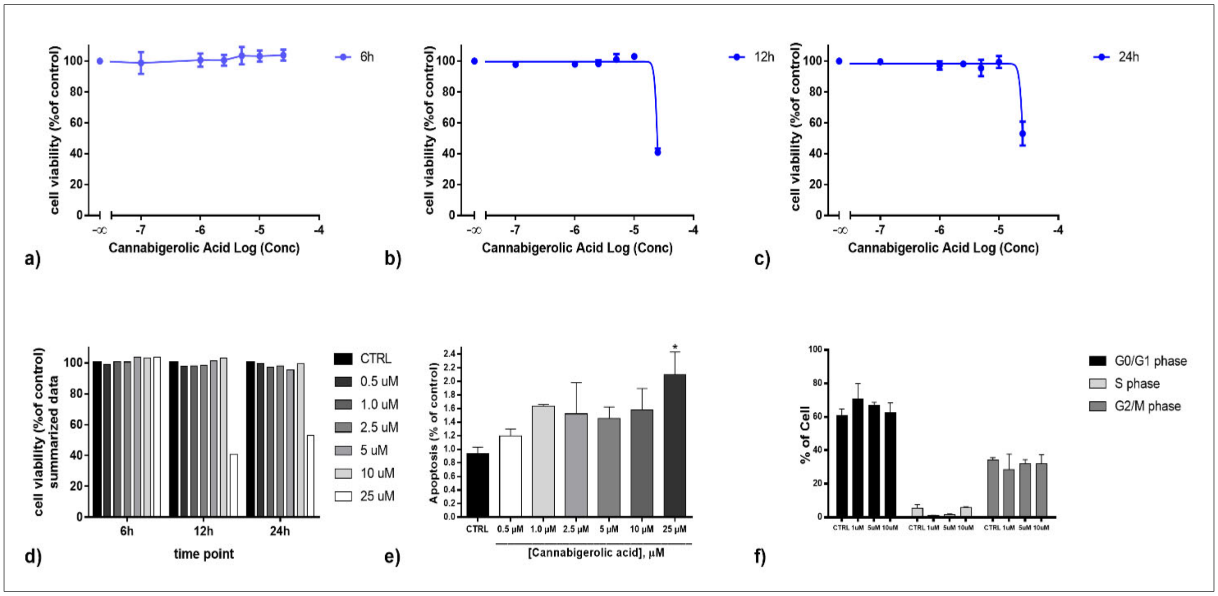

Figure S4. Viability, apoptosis, and cell cycle of human HaCaT cells treated with CBGA. Cell viability of HaCaT cells treated with vehicle (CTRL) or increasing concentrations of CBGA (0.5 μM, 1.0 μM, 2.5 μM, 5.0 μM, 10 μM, 25 μM), at the following time points: a) 6h, b) 12h, c) 24h. Values represent the mean ± SEM of three independent experiments. d) Summary of cell viability data shown in panels a-c. e) Apoptosis of HaCaT cells treated with vehicle (CTRL) or increasing concentrations of CBGA (0.5 μM, 1.0 μM, 2.5 μM, 5.0 μM, 10 μM, 25 μM) for 24h. f) Cell cycle analysis of HaCaT cells treated with vehicle (CTRL) or increasing concentrations of CBGA (1.0 μM, 5.0 μM, 10 μM) for 24h. Values represent the mean ± SEM of three independent experiments (n = 3). Statistical analysis was performed by ONE-WAY ANOVA test followed by Bonferroni *post hoc* test. *$P<0.05$ *vs* CTRL.

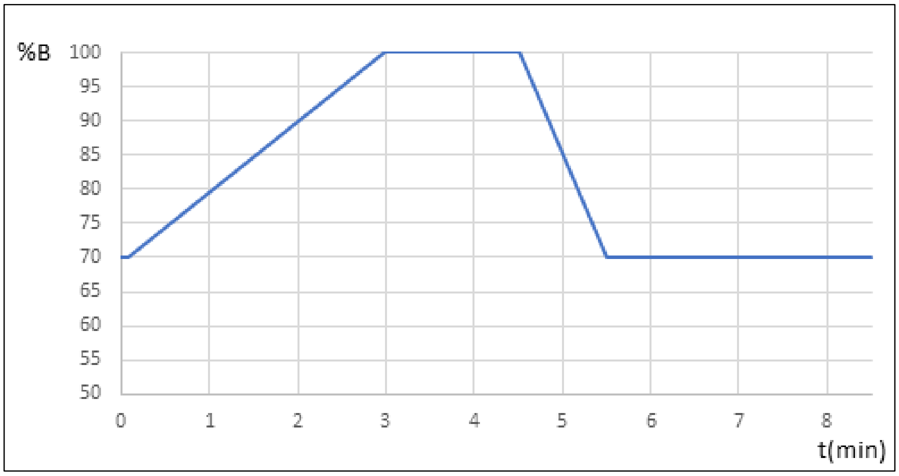

Figure S5. LC parameters. The chromatographic separation of target analytes was performed using a Kinetex XB-C18 1.7 μm (100x2.1 mm), with water 0.01% v/v of formic acid as mobile phase A and acetonitrile 0.01% v/v of formic acid as mobile phase B. The chromatographic gradient shown in the figure was applied for the LC-MS/MS analysis.

Table S1. MS/MS parameters. Parameters of LC-MS/MS method are reported: retention time (RT), limit of quantification (LOQ), precursor mass (Q1), declustering potential (DP), entrance potential (EP), fragment mass (Q3), collision energy (CE) and collision exit potential (CXP).

Compound	RT (min)	LOQ (pmol 10^6 cells)	Q1 (m/z)	DP (eV)	EP (eV)	Q3 (m/z)	CE (eV)	CXP (eV)
AEA	2.48	0.002	348.0	93.5	3.6	62.0	22.0	4.0
						287.1	21.0	14.0
AEA d8	2.48	-	356.1	93.5	3.6	62.0	22.0	4.0
2-AG	2.79	0.164	379.0	92.5	6.0	287.1	19.0	8.8
						269.0	23.0	10.0
2-AG-d8	2.79	-	387.2	92.5	6.0	294.6	19.0	8.8
PEA	2.86	0.004	300.1	97.7	7.0	62.0	21.0	4.1
						283.1	23.0	10.6
PEA-d4	2.86	-	304.1	97.7	7.0	62.1	21.0	4.1
